# Recurrent late-onset neutropenia after ofatumumab treatment in a case of multiple sclerosis

**DOI:** 10.1186/s42466-025-00377-0

**Published:** 2025-03-24

**Authors:** Jessy Chen, Thomas Burmeister, Lou Frankenstein, Inga Laumeier, Volker Siffrin

**Affiliations:** 1 Experimental and Clinical Research Center (ECRC), Charité-Universitätsmedizin Berlin und Max Delbrück Center or Molecular Medicine in the Helmholtz Association, Berlin, Germany; 2 Department of Neurology, Charité Universitätsmedizin Berlin, Berlin, Germany; 3 Department of Hematology, Oncology and Tumor Immunology, CVK, Charité- Universitätsmedizin, corporate member of Freie Universität Berlin and Humboldt-Universität zu Berlin, Berlin, Germany; 4 Department of Psychiatry and Neuroscience, Charité Universitätsmedizin Berlin, Berlin, Germany; 5 Neurological Practice (Neurologie und Neurochirurgie am Hackeschen Markt), Berlin, Germany

**Keywords:** B-cell therapy, Multiple sclerosis, Neutropenia

## Abstract

**Objective:**

Immunomodulatory treatment options for multiple sclerosis show an inverse risk‒benefit ratio of side effects and treatment efficacy. Although rare, anti-B-cell therapies can cause acute or late-onset neutropenia.

**Methods:**

We report a case of severe recurrent fluctuating neutropenia after ofatumumab treatment.

**Results:**

We observed four recurrences even after pausing with ofatumumab and repeated granulocyte stimulating factor (G-CSF) treatment. In total, neutropenia occurred five times and was associated with recurrent pulmonary, urinary tract, and skin infections. Bone marrow investigation revealed no signs of lymphoma or leukemia. Interestingly, routine molecular testing revealed two gene variants of unknown significance for *BCORL1* and *ASXL1*, both of which play a role in hematopoiesis. The neutrophil count recovered spontaneously six months after the cessation of treatment with ofatumumab.

**Discussion:**

This case highlights the necessity of identifying patients at risk and monitoring white blood cell counts regularly for up to 6 months after initial neutropenia.

## Introduction

Available treatment options for multiple sclerosis are associated with an inverse risk‒benefit ratio of side effects and treatment efficacy. Intravenous anti-CD20 agents (rituximab, ocrelizumab) carry an increased risk of neutropenia [1–3]. Similarly, this has been reported for the subcutaneous therapy option ofatumumab (OFA) [4–6]. The suggested mechanisms include a possible imbalance of granulopoiesis and neutrophil removal, e.g., growth factor remodeling might favor B-cell recovery or prolonged neutrophil removal due to drug-associated infections [7]. Case reports and reviews to date have documented both acute and late-onset neutropenia responsive to granulocyte colony-stimulating factor (G-CSF) treatment [1, 2]. Here, we present a severe case of OFA associated recurrent neutropenia, where neutropenia occurred in a cyclic pattern up to six months after the cessation of the treatment and required G-CSF treatment repeatedly.

### Case presentation

**Fig. 1:**
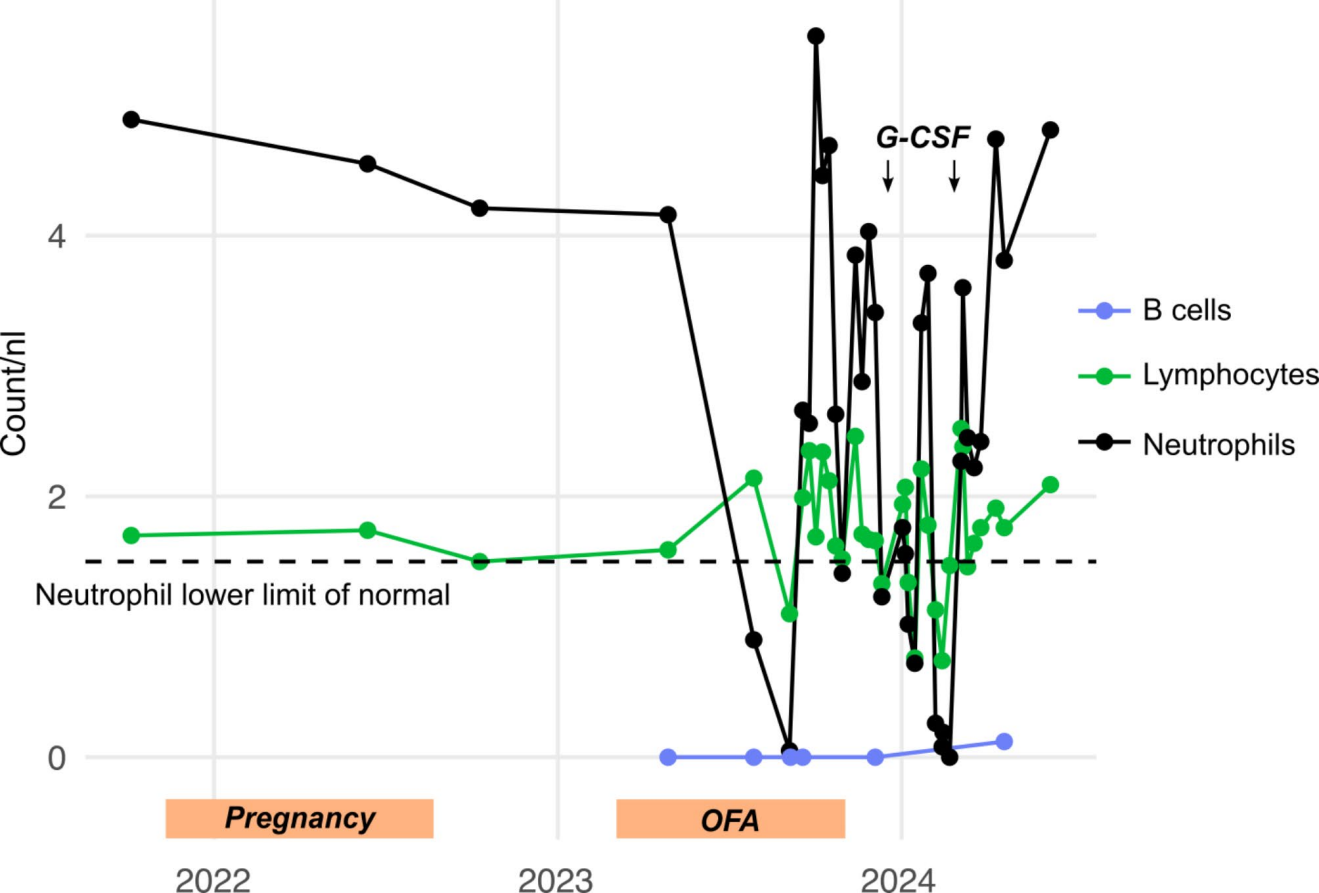
Overview of immune cell counts. The graph displays counts of lymphocytes, neutrophils, and B cells before, during and after the OFA treatment

We present the case of a 31-year-old female patient with relapsing-remitting MS who had an otherwise unremarkable medical history. She was first diagnosed with MS in 2021 following an episode of bilateral optic neuritis, meeting the 2017 revised McDonald criteria. Therapy with beta interferon (Plegridy^®^) was first initiated. Two months after MS diagnosis, the patient became pregnant and continued beta interferon throughout her pregnancy. However, follow-up magnetic resonance imaging (MRI) revealed newly formed spinal lesions, prompting escalation to anti-CD20 therapy (ofatumumab, OFA). This treatment began in February 2023, seven months postpartum. A routine blood test conducted by her external neurologist four months after starting OFA indicated reduced neutrophil levels (0.9/nl) in July 2023. Shortly after, the patient reported to our emergency room with pain around the left eyebrow and cheek. No clinical or laboratory signs of infection were observed at that time. In September 2023, she developed a tongue abscess requiring surgical intervention and antibiotic therapy. In the blood tests, neutropenia (0.51/nl) was diagnosed. She received antibiotic treatment with ampicillin/sulbactam for three days. Her neutropenia spontaneously resolved within two weeks. Initially, we opted to continue OFA treatment with weekly neutrophil monitoring due to the patient’s prior use of metamizole, which we considered a potential alternative cause of neutropenia. Two months later, neutropenia recurred, prompting discontinuation of OFA in October 2023. Spontaneous recovery was followed by additional severe recurrences in mid-December (0.0/nl), mid-January (0.72/nl), and February (0.03/nl), demonstrating an almost cyclic pattern (Fig. 1). Over this period, she received G-CSF twice, which did not induce MS relapse activity. Consultation with the hematology department included a bone marrow analysis, which revealed no signs of malignancy and normal cellularity with left-shifted, maturing granulopoiesis up to the promyelocyte stage. Segmented granulocytes were absent, and only a few band forms were visible. We did not observe pronounced lymphocyte fractions or T-cell large granular lymphocytes. Routine molecular screening for myeloid and lymphoid neoplasms identified two gene variants in *BCORL1* and *ASXL1*, classified as variants of unknown significance (VUS). The variant allele frequency (VAF) was nearly 50%, and germline status was confirmed via genetic analysis of a buccal swab. Further details on these variants are provided in Table 1. The patient has since undergone regular blood monitoring and has remained clinically stable. Notably, she became pregnant before initiating an alternative MS treatment. Natalizumab was identified as the preferred treatment option moving forward.

**Table 1:** Detailed information on gene variants in *BCORL1* and *ASXL1* genes

Gene	Variant description	Genomic coordinates (GRCh37)	Mutation	Allele frequency (global)	Protein change	Clinical relevance	ID
ASXL1	c.2110G > T	20:31022625	missense	0.000010	Missense variant	Unclear	rs151317625
BCORl1	c.3089G > A	X:129,149,837	missense	unknown	unclear	Unclear	COSM8501104

## Discussion

This case illustrates a rare but severe side effect of subcutaneous anti-CD20 therapy in line with a recently published case report by Protopapa and colleagues [8] and previous report with intravenous anti-CD20 therapies [1]. Notably, our case demonstrates that recurrent neutropenia can occur even in the absence of prior intravenous anti-CD20 therapy. Hitherto, neutropenia following subcutaneous OFA seemed to be less common (ca. 0.3%) than following ocrelizumab (ca. 4.5%) [3, 5]. Given the rarity of reported recurrent neutropenia in intravenous applications [1, 7, 9], it is crucial to investigate whether subcutaneous B-cell depletion agents are more frequently associated with this complication. Routine hematological diagnostics in our case identified two heterozygous germline gene variants in *BCORL1* and *ASXL1*. Both genes have previously been linked to various myeloid neoplasms and may play a role in neutropoiesis [10, 11]. However, further analysis of databases such as gnomAD, ClinVar, and COSMIC provided limited insights. The *BCORL1* variant was cited in only one COSMIC reference, while the clinical significance of the *ASXL1* variant remains unclear. It might be of interest to screen more patients experiencing neutropenia after anti-CD20 therapy for gene variants in *ASXL1* and *BCORL1*.

Our case report underscores that the subcutaneous application of anti-CD20 therapy can cause severe recurrent neutropenia. Clinical management should include regular blood monitoring for up to six months post-treatment to identify and mitigate potential complications.

## Data Availability

The datasets used and analyzed during the current study are available from the corresponding author upon reasonable request.

## Abbreviations

AML: Acute myeloid leukemia
CSF: Cerebral spinal fluid
G-CSF: Granulocyte colony-stimulating factor
IgG: Immunoglobulin G
MRI: Magnetic resonance imaging
MS: Multiple sclerosis
OFA: Ofatumumab
VAF: Variant allele frequency
